# Post-prandial analysis of fluctuations in the platelet count and platelet function in patients with the familial chylomicronemia syndrome

**DOI:** 10.1186/s13023-023-02743-0

**Published:** 2023-06-27

**Authors:** Miriam Larouche, Diane Brisson, Marie-Claude Morissette, Daniel Gaudet

**Affiliations:** Department of Medicine, Université de Montréal, ECOGENE-21, 930 Jacques Cartier Est, Chicoutimi, G7H 7K9 Canada

**Keywords:** Platelets, Familial chylomicronemia syndrome, Lipoprotein lipase, Lymphocytes, Pancreatitis, Volanesorsen

## Abstract

**Background:**

The familial chylomicronemia syndrome (FCS) is an ultra rare disease caused by lipoprotein lipase (LPL) deficiency associated with potentially lethal acute pancreatitis risk. Thrombocytopenia (platelet count < 150,000 × 10^9^/L) has been reported in patients with FCS, treated or not with volanesorsen, a second generation APOC3 anti-sense oligonucleotide. Chylomicrons are the lipoproteins delivering fat after a meal and FCS thus has a post-prandial origin. Platelet count and function have not been studied post-prandially in FCS.

**Objective:**

To evaluate post-prandial fluctuations in the platelet count (PLC) and functional defects of hemostasis in FCS.

**Methods:**

PLC, functional defects in hemostasis and hematologic variables were measured up-to 5 h after a meal in 6 homozygotes for FCS causing gene variants (HoLPL), 6 heterozygotes for LPL loss-of-function variants (HeLPL) and 7 normolipidemic controls.

**Results:**

Hourly post-prandial PLC was significantly lower in HoLPL than in controls (*P* < 0.009). Compared to the other groups, the PLC tended to decrease rapidly (in the first hour) post-meal in HoLPL (*P* = 0.03) and remained lower than baseline 5-h post-meal (*P* = 0.02) whereas it tended to slightly increase in normolipidemic controls (*P* = 0.02). Platelet function was not affected by the prandial status. In HoLPL, post-prandial fluctuations in the PLC positively correlated with the lymphocyte count (*P* = 0.005) and negatively with neutrophil/lymphocyte ratio (NLR).

**Conclusion:**

The PLC decreases post-prandially in FCS (HoLPL), is not associated with changes in functional defects of hemostasis and correlates with the NLR, a marker of acute pancreatitis severity.

## Introduction

Chylomicrons, the lipoproteins produced by the gut after a meal, are responsible for the transport and delivery of dietary triglycerides (TG) and cholesterol to peripheral tissues and to the liver. Once in circulation, chylomicron-TG are hydrolyzed by lipoprotein lipase (LPL) mostly produced in adipose and muscle tissues. In the event of a defect in LPL function or bioavailability, lipolysis is compromised, causing the accumulation of chylomicrons, which characterizes chylomicronemia. Chylomicrons are the largest circulating lipoproteins (> 100 nm), and plasma TG concentration remains elevated (> 10 mmol/L (890 mg/dl)), in patients with the familial chylomicronemia syndrome (FCS) (ORPHA:309,015), a rare autosomal recessive disorder characterized by sustained chylomicronemia due to bi-allelic combinations of loss-of-function (LoF) variants in the LPL gene or in genes coding for proteins essential to LPL efficacy and bioavailability, such as APOC2, GPIHBP1, APOA5 or LMF1 (1). Approximately 80% of FCS cases are explained by LoF mutations in the LPL gene (1). FCS represents a high risk of recurrent acute pancreatitis and other complications (2–5) and affected subjects poorly respond to fibrates and other currently used lipid lowering agents, although new therapeutic options, including APOC3 inhibitors are gradually emerging. Volanesorsen was a second generation APOC3 antisense oligonucleotide (ASO) (6–8). Although highly effective in decreasing TG, volanesorsen therapy was however associated with thrombocytopenia (platelet count below 150,000 × 10^9^/L), leading regulatory agencies to put in place risk mitigation strategies requiring frequent platelet count assessments. Although several elements suggest that volanesorsen-induced thrombocytopenia is related to the drug construct, recent data suggest that important fluctuations in the PLC is part of the natural history of FCS (9). It is documented in normolipidemic subjects that the PLC slightly increases after a meal without altering platelet function (10). FCS is the consequence of blood accumulation of chylomicrons, the large lipoproteins transporting fat after a meal. FCS has thus a post-prandial origin but the consequences are chronic, multisystemic and peiotropic. However, the PLC has not been studied post-prandially in patients with sustained chylomicronemia. The objective of the present study was thus to evaluate post-prandial fluctuations in the platelet count (PLC) and functional defects in hemostasis in FCS (HoLPL).

## Method

### Subjects and LPL status

A total of 19 patients were included in this study among which 6 subjects with sustained chylomicronemia carrying bi-allelic FCS causing mutations in the LPL gene (HoLPL), 6 heterozygotes for FCS complete or partial loss-of-function LPL gene mutations and history of chylomicronemia (HeLPL) and 7 normolipidemic wild-type controls (WT). FCS status was assessed by genotyping for FCS-causing mutations. The lipid profile, biochemical and hematological variables were measured by an accredited clinical laboratory using standardized and validated techniques. Multiparity Analyzer CX7 (Beckman) measured enzymatically cholesterol and TG levels. HDL level was measured after precipitation of the low-density lipoprotein (LDL) with heparin and MnCl_2_. None of the participants were taking a drug affecting bleeding or hemostasis, including low dose aspirin. No participant, in any group, was treated with a pharmacologic agent known to affect the PLC. No particpants took or had taken any APOC3 or ANGPTL3 inhibitor in the 12 months preceding the study including second (volanesorsen) or Galnac third generation (olezarsen) ASO, APOC3-iRNA, or ANGPTL3-iRNA or monoclonal antibody(mab).

All participants gave their informed consent. This study was conducted as part of the SMASH research program (SMASH: Systems and Molecular Approaches of Severe Hyperlipidemias) and was approved by IRB services (Advarra).

### Post-prandial hematological and platelet parameters

Hematological parameters, the lipid-lipoprotein profile, the PLC and platelet function (PFA) were assessed hourly up to 5 h following a meal standardly eaten at home by the participants. Thrombocytopenia was defined according to international standards as a PLC < 150 × 10^9^/L, whereas PLC > 450 × 10^9^/L was defined as thrombocytosis (11). Platelet function was measured with a Siemens PFA-100 by asssessing occlusion time on a collagen matrix coupled with epinephrine to activate platelet aggregation. Normal occlusion time ranges used were 65 to 150 s. The complete blood count (CBC) was assessed with a Beckman Coulter DxH 690 T system.

### Statistical analyses

Analyses were performed using non-parametric tests. Kruskal–Wallis were used to assess variations between FCS (HoLPL), HeLPL subjects and normolipidemic controls for continuous variables such as PLC, PFA, lipidic profiles and blood cell count. Friedman analyses were computed to assess intra-group variations and Spearman’s rank correlation tests to quantify the linear association between continuous variables. Results were considered significant when *P* < 0.05 (two-sided). All statistical analyses were performed with SPSS software (v.26; IBM, Armonk, NY, USA).

## Results

Table 1 presents baseline (pre-meal) characteristics of all participants. The wild-type group was younger than the others. Patients with FCS (HoLPL) were characterized by an history of sustained chylomicronemia, whereas patients in the HeLPL group had fluctuant TG levels but all presented an history of at least one documented episode of chylomicronemia (TG levels > 10 mmol/L). FCS presented higher fasting TG levels than the 2 other groups (*P* = 0.001) and were the only ones having had acute pancreatitis episodes (range: 2 to 84 episodes). All participants presented baseline PLC values in a normal range, although FCS and HeLPL had lower mean values than the normolipidemic controls. The majority of FCS and HeLPL patients had an history of thrombocytopenia (at least one PLC value < 150 × 10^9^/L in the medical file). No differences were observed in the mean platelet volume, platelet function and neutrophil count between the groups. However, FCS presented a significantly higher neutrophil/lymphocyte ratio (NLR) (*P* = 0.02) than the two other groups.

**Table 1:** Participants’ characteristics at baseline (pre-meal)

Characteristic	FCS (HoLPL)	HeLPL	Control Patients	*P*-value
Sex ratio (M/F)	2/4	4/2	0/7	0.036
Age (y), range	37–74	53–72	25–62	0.032
Chylomicronemia	Sustained	At least once	No	
Fasting TG (mmol/L) (range)	25.5 (15.4–35.3)	3.5 (2.5–4.7)	0.8 (0.6–1.3)	0.001
Platelet count (× 10^9^/L) (range)	182 (161–217)	177 (139–227)	258 (194–329)	0.006
Mean platelet volume (fL) (range)	9.35 (7.5–11)	9.25 (7.9–10.4)	8.39 (7.4–9.5)	NS
Platelet function (sec) (range)	171 (88–300)	121 (73–149)	105 (67–122)	NS
Lymphocyte count (× 10^9^/L) (range)	1.62 (0.8–3.3)	1.43 (1.2–1.7)	1.69 (1.2–2.3)	NS
Neutrophil count (× 10^9^/L) (range)	5.93 (2.4–9.7)	3.58 (2.1–6.8)	3.33 (2.7–3.8)	0.1
NLR (range)	3.97 (2.4–7.0)	2.44 (1.5–4.0)	2.04 (1.6–2.7)	0.02

*HoLPL* homozygous for loss-of-function variant in LPL gene, *HeLPL* heterozygous for loss-of-function variant in LPL gene, *TG* triglycerides, *NLR* neutrophil/lymphocyte ratio

**Fig. 1:**
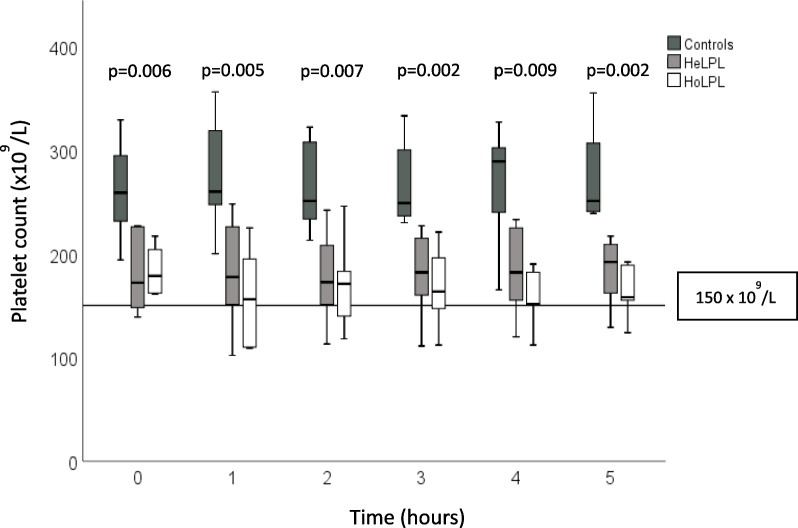
Hourly fluctuations of platelet values following a meal intake in FCS (HoLPL), HeLPL and normolipidemic controls. The line represents thrombocytopenia threshold. Hourly p-values compare differences in the PLC between normolipidemic controls vs the 2 other groups. In FCS, the PLC after 5 h was significantly lower than baseline (*P* = 0.021) which was not the case among HeLPL and controls (see text)

**Fig. 2:**
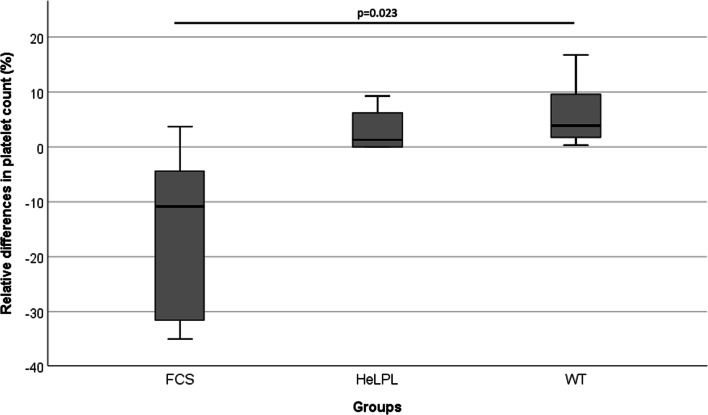
Relative post-prandial fluctuations (in %) 1 h after the standard meal in the platelet count in FCS (HoLPL), HeLPL and wild-type controls. FCS presented a significantly lower PLC and greater dispersion of values than HeLPL and WT controls (see text)

Figure 1 presents the fluctuations in the PLC from baseline up to 5 h post meal in each group. All platelet values remain in the normal range (> 150 × 10^9^/L) although they were significantly lower in FCS (HoLPL) and HeLPL patients compared to normolipidemic controls (*P* ≤ 0.009). The PLC tended to increase during the first hour post-meal in normolipidemic controls and remained stable afterwards. In contrast, the PLC decreased in the first hour in FCS (*P* = 0.02) and 5-h post-meal values were still lower than in the fasting state. When compared to the other groups, fluctuations in the PLC were also more pronounced in FCS (HoLPL) patients (Fig. 2) than in HeLPL or controls (*P* = 0.02). Figure 2 also shows that the PLC tended to decrease post-prandially in FCS and increase in normolipidemic subjects. Platelet function remained normal and stable in the three groups. Among other tested variables (data not shown), differences were noted in triglycerides levels and HDL-cholesterol concentration, TG values being higher and HDL-cholesterol lower in FCS compared to controls (*P* < 0.001) at each timepoint, which was expected considering the natural history of the disease (12).

**Fig. 3:**
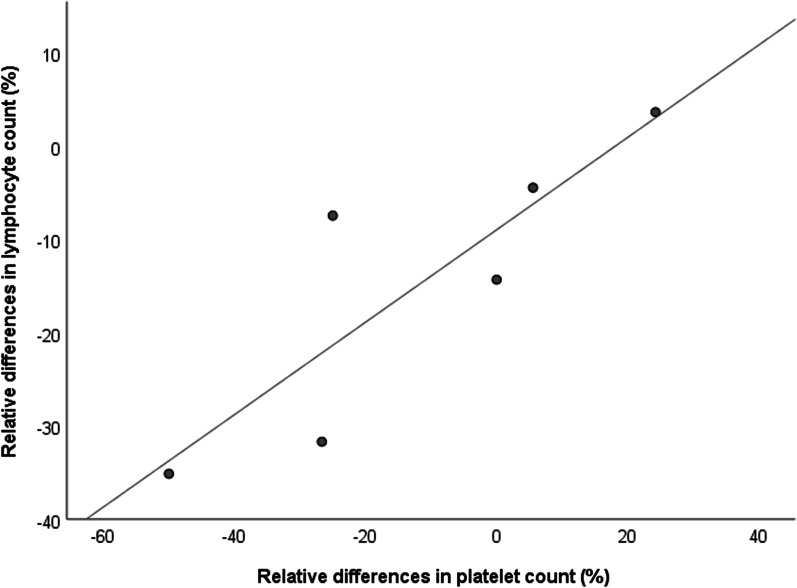
Correlation between relative differences in platelet and lymphocyte counts 1 h after the standard meal in FCS subjects

In the FCS (HoLPL) group, PLC relative fluctuations 1 h after a meal correlate with plasma TG (r_s_ = 0.9; *P* = 0.03), the lymphocyte count (r_s_ = 0.943; *P* = 0.005) and the neutrophil/lymphocyte ratio (r_s_ = − 0.829; *P* = 0.04). Figure 3 illustrates the correlation between post-prandial fluctuations in the lymphocyte count and the PLC in FCS (HoLPL) in the first hour.

## Discussion

Results of this study show that compared to normolipidemic controls and HeLPL, the PLC fluctuates and tends to decrease post-prandially in FCS (HoLPL). Post-prandial fluctuations in the platelet count are not associated with changes in platelet function but correlate with plasma triglyceride concentration, lymphocyte count and the neutrophil/lymphocyte ratio (NLR), a diagnostic marker of the severity of acute pancreatitis, an important feature of FCS (13, 14). One hour post-meal, the PLC decreased by 14% in FCS and slightly increased in normolipidemic controls an observation already made previously in healthy volonteers (10). Indeed, in a study involving 15 subjects, Wiens et al. observed a post-prandial increase in the PLC after a standard fat meal and demonstrated that platelet function was not altered by a meal (10). In their study, they measured collapsing time on an epinephrine soaked membrane at baseline, 3 h and 6 h following the meal intake. We were also able to demonstrate that platelet function was not altered by the meal, in all groups. The postprandial decrease in the PLC observed in FCS (HoLPL) seems paradoxal although it is compatible with the natural history of thrombocytopenia previously reported in these patients. It has indeed been previously documented that fluctuations in the PLC could be common in FCS (9). In a study reviewing PLC values collected over a 15-year period in 87 FCS (HoLPL) patients and 87 HeLPL, it has been observed that 55.2% of FCS presented a history of thrombocytopenia (PLC < 150,000 × 10^9^/L) on at least one occasion, 2.4% experiencing severe thrombocytopenia (PLC < 50 000 × 10^9^/L) (9).

Thrombocytopenia has also been observed in phase 2 and phase 3 clinical trials conducted with Volanesorsen in FCS. Volanesorsen is an antisense oligonucleotide (ASO) targeting APOC3 mRNA. It is a second generation ASO being currently replaced by a third generation Galnac-ASO having less effets on the PLC (olesarsen) (6).

During the phase 3 trial with volanesorsen (the APPROACH study) involving 66 FCS patients randomly assigned to receive the treatment or placebo in a 1:1 ratio, thrombocytopenia was observed in 25 treated patients (76%). The treatment was discontinued for two patients who had grade 4 thrombocytopenia (PLC < 25 × 10^9^/L). No other discontinuations were due to platelet decrease in this study (6). In the COMPASS study, volanesorsen was used to treat patients with multifactorial severe hypertriglyceridemia including a subgroup of 7 patients with FCS. Decrease in the PLC was observed in 9 patients (12%) treated and one on placebo. One patient was discontinued due to PLC decrease under 50 × 10^9^/L (15). It has been suggested that thrombocytopenia occured as the result of phosphorothioate modifications flanking the oligonucleotides to prevent rapid degradation by nucleases, a feature which is avoided with the development of the third generation APOC3 ASO olesarsen which uses Galnac technology for cell internalization and requires lower dosage than volanesorsen (16, 17).

Analysis of clinical trial data collected in a large database of studies using second generation ASO have been conducted. The effects of ASO therapeutics on PLC and function in more than 2600 subjects treated with 16 different 2’-O-methoxyethyl-modified ASOs (placebo-controlled and open label trials), including volanesorsen, were assessed. No significant decrease in platelet count was observed and less than 10 subjects (0.3%) presented a decrease in platelet count between 50 and 100 × 10^9^/L. No evidence of platelet activity alteration was observed or linked to treatment with ASOs neither (18, 19). A recent study suggests that Sequence-specific 2'-O-methoxyethyl antisense oligonucleotides activate human platelets through glycoprotein VI, triggering formation of platelet-leukocyte aggregates (20). Platelet activation by 2'MOE ASOs may cause increased expression of surface proteins, with platelet adhesion to neutrophils, and subsequent clearance of these cell complexes. However, this has not been specifically reported with volanesorsen. The more pronounced effect of volanesorsen on the PLC in patients with FCS than other diseases might reflect a combined effect of drug-induced and disease-induced phenomenon, since thrombocytopenia is likely part of the natural history of FCS, a post prandial disease. As a matter of fact, results of the present study suggest that the PLC decreases post-prandially in FCS and increases in the other groups similarly to that previously observed in healthy volonteers (18, 19). Gene expression analyses conducted in patients with FCS and thrombocytopenia suggest that more than 25% of genes are differentially expressed in presence of thrombocytopenia (21). A strong correlation was also observed between the platelets and lymphocytes counts in FCS (r_s_ = 0.943; *P* = 0.005). Platelets release different inflammatory mediators via their α-granules such as CD40L and CCL5 involved in immune-related cells recruitment, including lymphocytes (22).

FCS is a chronic post-prandial and pro-inflammatory disease. Chylomicrons are formed in the enterocytes and reach the bloodstream through the lymphatic system. If LPL is not or poorly available, the chylomicrons accumulate in the bloodstream and are subject to oxidative stress leading to a pro-inflammatory state (2), which is compatible with the correlation observed between the PLC and lymphocytes count in the HoLPL group. The platelet-lymphocyte ratio is a prognostic marker of inflammation, whereas the neutrophil/lymphocyte ratio (NLR) is a marker of acute pancreatitis severity (13, 14, 23). The correlation between the PLC and the NLR that has been observed in our study was mainly driven by the lymphocytes count. As shown in Table 1, FCS patients have a NLR higher than the other groups which is concordant with the risk of severe acute pancreatitis in presence of sustained chylomicronemia.

In this study, lower fasting (pre-meal) PLC was observed in patients with partial (HeLPL) or complete (HoLPL) LPL deficiency compared to controls but platelet hemostatic or coagulation effect was not affected. Fluctuations in the PLC and thrombocytopenia is part of the natural history of FCS (HoLPL) and has also been observed in a lesser extent among patients with partial LPL deficiency (HeLPL) (9). The important fluctuations in the PLC observed over time and decades in FCS suggest the involvement of platelets' characteristics not related to hemostasis, such as cargoing capability of micro particles or microRNA or contribution to immune and/or inflammatory responses. These characteristics may contribute to the FCS phenotype and clinical expression. Thrombocytopenia and platelets characteristics should be studied more extensively both post-prandially and in the fasting state in FCS (HoLPL). Such studies are ongoing and include functional analyses, gene expression profilling studies, exome sequencing, epigenetic and lipidomic studies. The present study has other limitations. First, the sample size is small, due to the rarity of the disease. With a prevalence of 1–2 per million (24), FCS is an ultra-rare disease. However, the fine phenotyping of patients having participated to this study and their tight follow-up for decades is quite unique. Results of this study require replication in a larger and more diversified cohort.

## Conclusion

FCS is a rare post-prandial disease associated with increased pancreatitis risk. Platelets functionality is not altered in FCS although significant post-prandial fluctuations in the PLC are noticed. Fluctuations in the PLC in FCS are thus not solely related to volanesorsen treatment and are likely part of the natural history of the disease. Post-prandial flcutuations in the PLC in FCS correlate with the NLR, a marker of acute pancreatitis severity.

## Data Availability

All data and details on methods and/or materials are available upon request.

## Abbreviations

APOA5: Apolipoprotein A5
APOC2: Apolipoprotein C2
APOC3: Apolipoprotein C3
ASO: Antisense oligonucleotide
CCL5: C-C motif chemokine ligand 5
CD40L: CD40 ligand
FCS: Familial chylomicronemia syndrome
GPIHBP1: Glycosylphosphatidylinositol anchored hidh density lipoprotein binding protein 1
HeLPL: Heterozygote carrier for loss of function LPL gene variant
HDL: High-density lipoprotein
HoLPL: Homozygote for loss of function LPL gene variant
LDL: Low-density lipoprotein
LMF1: Lipase maturation factor 1
LoF: Loss of function
LPL: Lipoprotein lipase
mRNA: Messenger RNA
NLR: Neutrophil/lymphocyte ratio
PFA: Platelet function
PLC: Platelet count
SMASH: Systems and molecular approaches of severe hyperlipidemia
TG: Triglycerides
WT: Wild-type

## References

[1] Blom DJ, O'Dea L, Digenio A, Alexander VJ, Karwatowska-Prokopczuk E, Williams KR (2018). Characterizing familial chylomicronemia syndrome: baseline data of the APPROACH study. J Clin Lipidol.

[2] Brunzell JD, Deeb SS. Familial Lipoprotein Lipase Deficiency, Apo C-II Deficiency, and Hepatic Lipase Deficiency. In: Valle DL, Antonarakis S, Ballabio A, Beaudet AL, Mitchell GA, editors. The Online Metabolic and Molecular Bases of Inherited Disease. New York: McGraw-Hill Education; 2019.

[3] Patsch JR, Miesenbock G, Hopferwieser T, Muhlberger V, Knapp E, Dunn JK (1992). Relation of triglyceride metabolism and coronary artery disease. Studies in the postprandial state. Arterioscler Thromb..

[4] Karpe F, Steiner G, Uffelman K, Olivecrona T, Hamsten A (1994). Postprandial lipoproteins and progression of coronary atherosclerosis. Atherosclerosis.

[5] Masuda D, Yamashita S (2017). Postprandial hyperlipidemia and remnant lipoproteins. J Atheroscler Thromb.

[6] Witztum JL, Gaudet D, Freedman SD, Alexander VJ, Digenio A, Williams KR (2019). Volanesorsen and triglyceride levels in familial chylomicronemia syndrome. N Engl J Med.

[7] Gaudet D, Alexander VJ, Baker BF, Brisson D, Tremblay K, Singleton W (2015). Antisense inhibition of apolipoprotein C-III in patients with hypertriglyceridemia. N Engl J Med.

[8] Gaudet D, Brisson D, Tremblay K, Alexander VJ, Singleton W, Hughes SG (2014). Targeting APOC3 in the familial chylomicronemia syndrome. N Engl J Med.

[9] Gaudet D, Baass A, Tremblay K, Brisson D, Laflamme N, Paquette M (2017). Natural history (up to 15 years) of platelet count in 84 patients with familial hyperchylomicronemia due to lipoprotein lipase deficiency. J Clin Lipidol.

[10] Wiens L, Lutze G, Luley C, Westphal S (2007). Platelet count and platelet activation: impact of a fat meal and day time. Platelets.

[11] Greenberg EM, Kaled ES (2013). Thrombocytopenia. Crit Care Nurs Clin North Am..

[12] Hayden MR, Ma Y (1992). Molecular genetics of human lipoprotein lipase deficiency. Mol Cell Biochem.

[13] Azab B, Jaglall N, Atallah JP, Lamet A, Raja-Surya V, Farah B (2011). Neutrophil-lymphocyte ratio as a predictor of adverse outcomes of acute pancreatitis. Pancreatology.

[14] Jeon TJ, Park JY (2017). Clinical significance of the neutrophil-lymphocyte ratio as an early predictive marker for adverse outcomes in patients with acute pancreatitis. World J Gastroenterol.

[15] Gouni-Berthold I, Alexander VJ, Yang Q, Hurh E, Steinhagen-Thiessen E, Moriarty PM (2021). Efficacy and safety of volanesorsen in patients with multifactorial chylomicronaemia (COMPASS): a multicentre, double-blind, randomised, placebo-controlled, phase 3 trial. Lancet Diabetes Endocrinol.

[16] Reeskamp LF, Tromp TR, Stroes ESG (2020). The next generation of triglyceride-lowering drugs: will reducing apolipoprotein C-III or angiopoietin like protein 3 reduce cardiovascular disease?. Curr Opin Lipidol.

[17] Flierl U, Nero TL, Lim B, Arthur JF, Yao Y, Jung SM (2015). Phosphorothioate backbone modifications of nucleotide-based drugs are potent platelet activators. J Exp Med.

[18] Crooke ST, Baker BF, Witztum JL, Kwoh TJ, Pham NC, Salgado N (2017). The effects of 2'-O-methoxyethyl containing antisense oligonucleotides on platelets in human clinical trials. Nucleic Acid Ther.

[19] Crooke ST, Liang XH, Baker BF, Crooke RM (2021). Antisense technology: a review. J Biol Chem.

[20] Slingsby MHL, Vijey P, Tsai IT, Roweth H, Couldwell G, Wilkie AR (2022). Sequence-specific 2'-O-methoxyethyl antisense oligonucleotides activate human platelets through glycoprotein VI, triggering formation of platelet-leukocyte aggregates. Haematologica.

[21] Tremblay K, Brisson D, Gaudet D (2017). Gene expression signature of platelet count in lipoprotein lipase deficiency. J Clin Lipidol.

[22] Thomas MR, Storey RF (2015). The role of platelets in inflammation. Thromb Haemost.

[23] Balta S, Ozturk C (2015). The platelet-lymphocyte ratio: a simple, inexpensive and rapid prognostic marker for cardiovascular events. Platelets.

[24] Ramasamy I (2018). Update on the laboratory investigation of dyslipidemias. Clin Chim Acta.

